# Transplantation of Amniotic Membrane to the Subretinal Space in Pigs

**DOI:** 10.1155/2012/716968

**Published:** 2012-03-05

**Authors:** Jens Folke Kiilgaard, Erik Scherfig, Jan Ulrik Prause, Morten la Cour

**Affiliations:** ^1^Department of Ophthalmology, Glostrup Hospital, Copenhagen University Hospital, Nordre Ringvej 57, 2600 Glostrup, Denmark; ^2^Eye Pathology Institute, Teilumbygningen, University of Copenhagen, 2100 Copenhagen, Denmark

## Abstract

*Purpose*. To investigate the effect of transplanted amniotic membrane (AM) on subretinal wound healing. *Methods*. Nine Danish Landrace pigs had surgical removal of retinal pigment epithelium (RPE) and mechanical damage of Bruch's membrane (BM) and served as a control group. 15 pigs additionally had AM transplanted to the subretinal space. *Results*. AM significantly reduces choroidal neovascularisation when complete coverage of the induced defect is obtained (7 pigs) (*P* < 0.05). In cases where AM did not cover the rupture in BM choroidal tissue covered the transplanted membrane (8 pigs). AM is well tolerated in the subretinal space, causes only limited inflammation, and is covered with a monolayer of pigmented cells when in contact with the host RPE. *Conclusions*. AM modifies choroidal neovascularisation after BM damage and may serve as a basement membrane substitute for the RPE.

## 1. Introduction

During the last decade there has been an immense progress within the field of study of retinal progenitor cells and today several clinical transplantation studies are ongoing (http://clinicaltrials.gov/). While the basic clinical studies often seek to prevent damage in either healthy animal injury models [1] or in models of retinal degeneration [2], the clinical situation often represents the end-stage situation with loss of cells and fibrotic scar formation. In this situation, survival of transplanted cells may be impaired.

A recent study, where retinal pigment epithelial (RPE) was transplanted into the subretinal space after CNV membranectomy, showed that the transplanted RPE was severely impaired by the still underlying active age-related macular degeneration (AMD) [3]. CNV membranes in exudative AMD eyes do not differ histologically from CNV membranes found in eyes with other underlying diseases, indicating that CNV membranes are a nonspecific wound repair to breaks in Bruch's membrane (BM) [4–6]. This may suggest that the entire milieu needs to be altered before transplanted cells can rescue retinal function [7]. Several synthetic materials have been tested as scaffolds for retinal progenitor cell transplantation and have been found to support adhesion, survival, and migration [8, 9].

We have previously shown that amniotic membrane transplanted into the subretinal space in young pigs stimulates the host RPE to cover this basement membrane substitute [10, 11]. The amniotic membrane (AM) is a basement membrane with antiangiogenic and anti-inflammatory properties well known in anterior segment reconstructive surgery. We have previously described a model of CNV in the pig [12, 13]. We wanted to investigate if the AM transplanted to the subretinal space in this model could facilitate regrowth of RPE cells and prevent the formation of a CNV membrane.

## 2. Material and Methods

Domestic pigs (Danish Landrace), 4 months old and weighing approximately 30 kg, were used as experimental animals. Their treatment followed the ARVO resolution for the use of animals in ophthalmic and vision research, and was supervised by a veterinarian nurse. The Danish Animal Experiments Inspectorate granted permission for the use of pigs in this study.

### 2.1. Amnion Membrane Isolation

 Porcine amniotic membranes from placenta from full borne pigs (114 days) and from 104-day-old foetuses were used. Placentas from full borne specific pathogen-free (SPF) pigs were obtained from the local supplier of SPF pigs. Placentas from foetuses were obtained by planned Caesarean section on SPF pigs. The amnionchorion-endometrium was identified and isolated. Using microscope and forceps it was possible to separate the AM from the chorion. Epithelial cells were removed from the AM by mechanically scraping with a silicone-coated tip after 15–20 min incubation in 0,1 M NH_4_OH followed by repeated freezing and thawing until histopathology confirmed complete removal of epithelial cells.

### 2.2. Anaesthesia

 All animals were preanaesthetised with intramuscular injections of 15 mg midazolam (Dormicum, Roche, Hvidovre, Denmark) and 250 mg ketamine (Ketalar, Parke-Davis, ballerup, Denmark) followed by intravenous administration of 250 mg mebumal (Mebumal, SAD, Copenhagen, Denmark). The pigs were endotracheally intubated, artificially ventilated, and anaesthetised with halothane 1% in combination with 66% NO2 in oxygen.

### 2.3. Induction of CNV

 Mechanical induction of a CNV has previously been described [13]. In short, a three-port localized vitrectomy was used. Access to the subretinal space was obtained with a retinal perforator (Synergetics Inc, st Charles, MO, USA). Retina was then carefully lifted off by injecting isotonic NaCl through a 30G needle (BSS injection needle, straight 1270, DORC international B.V., NL). An automatic scissor (STORZ, Bausch & Lomb Surgical, Inc., San Dimas, CA, USA) was used to enlarge the retinotomy before the RPE was gently removed using a retinal scraper (1294, DORC international B.V., NL). The subretinal space was then washed with isotonic NaCl through a 21G needle (number 5021, Visitec, FL, USA) to ensure total removal of damaged RPE, and finally rupture to BM was made with the retinal perforator.

### 2.4. Transplantation Technique

**Figure 1:**
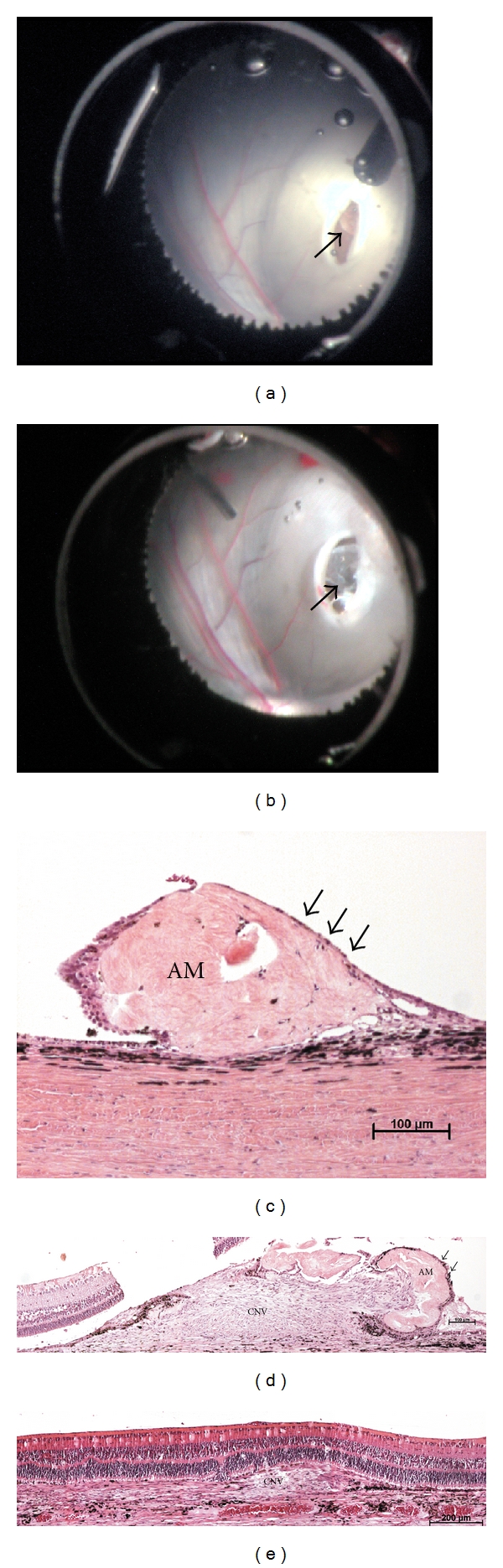
(a) Peroperative photo of the bleb with the retinotomy. Arrow points at the lesion in the retinal pigment epithelium trough the retinotomy. (b) Peroperative photo from the same operation as (a). The arrow points now at transplanted amniotic membrane (AM) covering the induced lesion. (c) Micrograph of the subretinal space (SRS) in a pig after transplantation of AM. The AM is in close contact with the RPE and covers the lesion completely. AM is totally covered with RPE cells similar to the normal host RPE and no CNV is observed. (d) Micrograph of the SRS in a pig after transplantation of AM. AM covers in this case only partly the induced lesion. Formation of a choroidal neovascularisation (CNV) is seen. AM is in contact with the RPE at one side and the RPE has covered the retina near side of the AM. We have not observed CNV penetrate the AM, but the CNV rather tends to “crawl under” the membrane out into the SRS. (e) Micrograph of a control eye that received mechanical damage to RPE/Bruch's membrane, but did not receive any AM. A large CNV is seen in the SRS. The peripheral part is covered with RPE, whereas the central part is in close contact with the photoreceptors. The scale bars are: (a) 100 *μ*m, (b) 100 *μ*m, and (c) 200 *μ*m.

AM was transplanted to the SRS in 15 pigs in addition to the CNV induction. DORC's Combined Spatula/Peeling Forceps (1297, DORC international B.V., NL) was used to deliver the AM to the SRS. The DORC hockeystick Microforceps (1286 LFT, DORC international B.V., NL) was then used to flatten the AM over the RPE lesion in the SRS (Figures 1(a) and 1(b)).

### 2.5. Followup

The observation period varied between 14 and 42 days. Prior to enucleation the pigs were examined by ophthalmoscopy and fundus photography. All pigs were hereafter euthanized by a lethal injection of 4 g pentobarbital with 400 mg of lidocaine hydrochloride (Veterinærapoteket Københavns Universitet, Copenhagen, Denmark).

### 2.6. Histopathology

 Enucleated eyes were immediately fixed in 4% buffered formaldehyde (Lilly's fluid, Bie & Berntsen, Rødovre, Denmark) overnight at room temperature and paraffin embedded according to routine procedures. Sections of 4 *μ*m were cut and stained with haematoxylin and eosin. Haematoxylin/phloxine/saffron staining was used to identify transplanted amnion membrane. Induced inflammation in the adjacent retina and choroid was scored semiquantitatively based on the number of lymphocytes per visual field (×10 magnification) (0 = 0–10; 1 = 10–100; 2 = 100–1000; 3 >1000). In the analysis of the data the groups could be reduced further to 0: no increase and 1: inflammation (>100/field view).

### 2.7. Measurement of CNV

 All sections were examined with a light microscope, Axioplan 2 (Carl Zeiss, Jena, Germany); digital images were obtained with an Axiocam HRC (Carl Zeiss). Areas of interest were demarcated on digital images and their sizes were computed with the software package Axiovision 3.1 (Carl Zeiss). Average CNV thickness was preferred as a size measurement because it has proved to be the most reproducible size measurement at our disposal in this model [12]. Kruskal-Wallis one-way analysis of variance on ranks was performed. Statistics and graphs were made in SigmaStat/SigmaPlot (Systat Software Inc., San Jose, CA).

## 3. Results

In this study, a total number of 24 pigs were induced with CNV. 15 pigs were transplanted with AM after mechanical removal of RPE and rupture of BM. 9 pigs served as a control group. The control group has been described histologically in a previous study [13].

**Figure 2:**
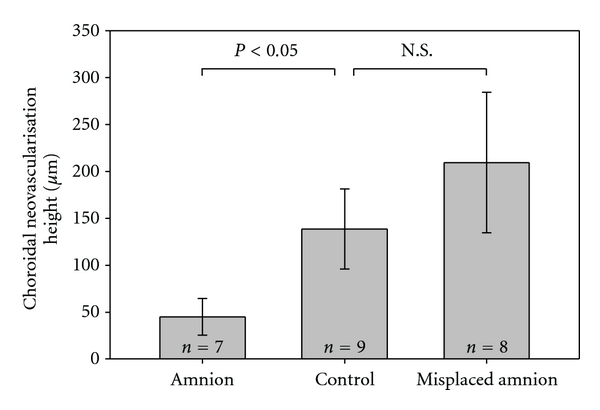
Graph of maximal height in induced choroidal neovascularisation measured in um. Experiments were divided in successful (amnion), unsuccessful (misplaced amnion), and control (no amnion). Data are presented as mean +/− SD. Kruskal-Wallis one-way analysis of variance on ranks was performed.

The lesion in BM and the induced CNV could be identified in all 24 eyes. In 7 eyes, AM was found to completely cover the BM lesion. In 8 eyes, AM did not cover the entire lesion, and in 3 of these cases AM was not in contact with the induced defect in BM. The measurements of the CNV are given as mean +/− SD in Figure 2.

### 3.1. Surgery

 The lens protrudes far back into the vitreous body of pigs making the lens vulnerable to peroperative damage. However, surgical complications after transplantation of amniotic membranes were limited. Four pigs had localised cataracts close to upper nasal sclerotomy most likely induced by the retinal perforator. No pigs were excluded due to surgical complications.

### 3.2. Inflammatory Response

 The induction of CNV in the pig induces a huge inflammatory response in the main choroid but also the retina. This inflammation in the choroid is seen in 100% of the control group. The transplanted AM reduced the number of inflammatory cells in the tissue close to the transplanted membrane material (data not shown). In the cases with successful placed AM and minimal CNV no increase in number of inflammatory cells was observed. This difference was significant (*P* < 0.005, Fischer's exact test). Inflammation in the neuroretina was, however, comparable between the two groups (control: 70%, AM: 34%, *P* = 0.28).

### 3.3. RPE Response

 The transplanted amniotic membranes were completely or partly covered with RPE cells. The RPE was always observed as a monolayer on the inner surface of AM. The pigmentation of the RPE varied from completely depigmented to normal pigmentation with cigar-shaped granules. Depigmented RPE cells on the AM were always found in combination with a zone of depigmented RPE cells on normal-looking BM surrounding the AM. Such a zone with depigmented RPE cells on BM was never observed in cases where normal pigmented RPE cells had covered the AM.

## 4. Discussion

In this paper we demonstrate that isolated amniotic membrane can modify the formation of choroidal neovascularisation in a porcine model of subretinal wound healing. Amniotic membrane is a well-known inhibitor of neovascularisation on the ocular surface [14], and anti-inflammatory and antiangiogenic proteins have not only been shown to be synthesised in the amniotic epithelium, but they have also been identified in the compact layer of the AM stroma [15]. We do observe a few new vessels within the membrane, but these vessels are only found in the space beneath the membrane and are never observed to penetrate the AM. The fact that we only used AM without epithelium for transplantation and the observed short radius of action makes it likely that the anti-inflammatory and antiangiogenic effect of the AM are dependent on factors in the stroma, and that these factors are immobilised in the membrane. Decreased, or lack of, inflammation was also observed in tissue in close contact to the AM.

The idea of delivery of active drugs together with cellular transplants is a major objective in the construction of synthetic scaffolds [16], but AM may happen to be a natural reservoir for relevant growth factors for the optimal survival for stem cells. Increased attention on AM as a basement membrane support for cultured RPE is therefore seen in vitro [17, 18] and in vivo [10, 11].

AM is, in contrast to the anterior les capsule (ALC), another basement membrane [19], very easy to handle and flatten in vitro. In vivo we succeeded to flatten the AM in the subretinal space during surgery (Figure 1(b)), but unfortunately, histology show that we were not able to maintain the AM flattened in the SRS (Figures 1(c) and 1(d)). We are currently evaluating different techniques that could circumvent the problem. The use of gas or silicone oil could as well keep retina and the underlying AM flat, but may also cause increased glial reaction on the naked AM as seen when neuroretina gets into contact with naked ALC [19]. Use of photocoagulation to weld the AM to the underlying BM might be another possibility. However, scar formation takes time and photocoagulation may therefore need to be used in combination with a method that keeps the AM in place while scarring occurs. Bioadhesives like the types used to glue retinal prosthesis to the retina are being developed [20] and might be a more suitable solution.

The orientation of the AM in the SRS might be important, but we did not test for this in this study. The AM is part of an organised structure and is in itself a polarised membrane. Only the surface toward the foetus is covered with epithelium. We did not harvest from a specific part of the yolk sack and we did not check the orientation of the transplanted membranes. More careful harvesting procedures and storage of the membranes on filter paper to allow orientation of the membrane could answer these questions in future studies.

The ingrowths of RPE on the membrane support our previous observations in both ALC transplantations and RPE removal experiments [10, 19]. RPE cells covering denuded BM become more and more flat and depigmented the farther away they are from intact RPE whereas RPE covering the AM maintains morphology similar to adjacent RPE. Therefore, it seems important not only to cover the rupture itself in BM, but also to cover the denuded BM placing the AM in immediate contact with healthy RPE.

The AM seems to be a good candidate for future studies, since this membrane can be handled surgically and has anti-inflammatory and antiangiogenic properties that enable the correctly placed AM to inhibit the formation of CNV. Finally, the AM is an excellent growth support for cells whatever host RPE cells, new RPE cells, or stem cells are needed in the SRS.

## Abbreviations

AM: Amniotic membrane
ALC: Anterior lens capsule
BM: Bruch's membrane
CNV: Choroidal neovascularisation
H&E: Haematoxylin and eosin
RPE: Retinal pigment epithelium
SD: Standard deviation.
